# Pancreatic cancer‐derived small extracellular vesical ezrin activates fibroblasts to exacerbate cancer metastasis through STAT3 and YAP‐1 signaling pathways

**DOI:** 10.1002/1878-0261.13442

**Published:** 2023-05-12

**Authors:** Yu‐Ting Chang, Hsuan‐Yu Peng, Chun‐Mei Hu, Sui‐Chih Tien, Yi‐Ing Chen, Yung‐Ming Jeng, Ming‐Chu Chang

**Affiliations:** ^1^ Department of Internal Medicine, College of Medicine National Taiwan University Taipei Taiwan; ^2^ Department of Internal Medicine National Taiwan University Hospital Taipei Taiwan; ^3^ Genomics Research Center Academia Sinica Taipei Taiwan; ^4^ Department of Pathology, College of Medicine National Taiwan University Hospital, National Taiwan University Taipei Taiwan

**Keywords:** cancer‐associated fibroblasts, ezrin, metastasis, pancreatic ductal adenocarcinoma, small extracellular vesicles

## Abstract

Cancer‐associated fibroblasts (CAFs), a major component of the tumor microenvironment (TME) in pancreatic ductal adenocarcinoma (PDAC), play an important role in tumorigenesis, metastasis, and chemoresistance. Tumor‐derived small extracellular vesicles (sEVs), which mediate cell‐to‐cell communication between cancer cells and fibroblasts, are also critical for cancer progression and metastasis. However, it remains unclear how PDAC cell‐derived sEVs activate fibroblasts, which contributes to tumor progression. Here, we report that ezrin (EZR) expression in PDAC cell‐derived sEVs (sEV‐EZR) can activate fibroblasts, resulting in increased migration ability and high expression of α‐SMA, PDGFRB, and high production of extracellular matrix in fibroblasts. Reciprocally, sEV‐EZR‐activated fibroblasts enhanced PDAC cell proliferation, invasion, and metastasis to the liver in animal models. Conversely, fibroblasts treated with PDAC cell‐derived sEVs with EZR knockdown resulted in the reduced metastatic ability of PDAC. Mechanistically, we demonstrated that PDAC cell‐derived sEV‐EZR increases the STAT3 and YAP‐1 signaling pathways to induce fibroblast activation, and the activated fibroblasts promote PDAC cell proliferation, invasion, and liver metastasis. Inhibition of the STAT3 and YAP‐1 signaling pathways by gene knockdown can abrogate sEV‐EZR‐induced effects. These findings suggest that targeting the interaction between PDAC cell‐derived sEV‐EZR and fibroblasts is a potential therapeutic strategy for PDAC.

## Graphical abstract

PDAC cell‐derived sEVs can activate fibroblasts through activating STAT3 and YAP‐1 signaling, resulting in increased migration ability and high expression of α‐SMA, PDGFRB, and high production of extracellular matrix in fibroblasts. Reciprocally, sEV‐EZR‐activated fibroblasts enhanced PDAC cell proliferation, invasion, and metastasis to the liver. Inhibition of the STAT3 and YAP‐1 signaling pathways can abrogate sEV‐EZR‐induced effects. Thus, targeting sEV‐EZR may contribute to improve adjuvant PDAC therapies.

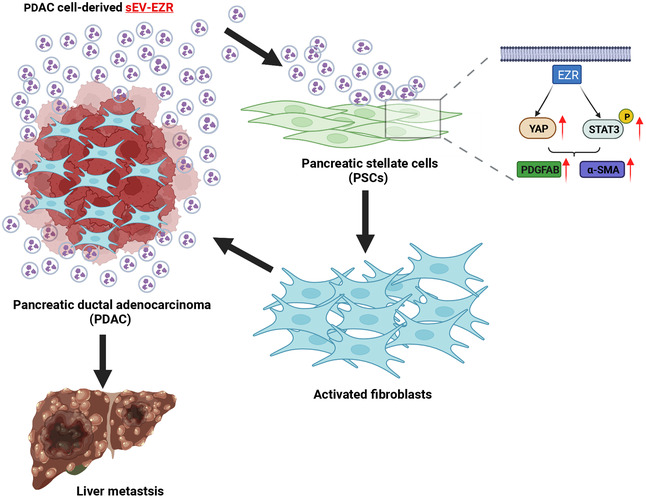

**Abbreviations**
CAF: cancer‐associated fibroblasts
ECM: extracellular matrix
ERM: ezrin/radixin/moesin
EZR: ezrin
FAK: focal adhesion kinase
FAP: fibroblast activation protein
HPaSteC: human pancreatic stellate cells
HPDE: human pancreatic ductal epithelial cells
HPNE: nontransformed human pancreatic acinar‐to‐ductal epithelial‐like cells
IHC: immunohistochemistry
IL‐6: interleukin‐6
JAK: janus kinase
Lip: liposome
miR: microRNA
MSCs: mesenchymal stem cells
NF‐κB: nuclear factor kappa‐light‐chain‐enhancer of activated B
NSG: NOD/SCID/IL2Rγnull
PDAC: pancreatic ductal adenocarcinoma
PDGFRA: platelet‐derived growth factor‐receptor A
PDGFRB: platelet‐derived growth factor‐receptor B
PI3Ks: phosphoinositide 3‐kinases
PSCs: pancreatic stellate cells
sEVs: small extracellular vesicles
shEZR: short hairpin RNA ezrin
shLacz: short hairpin RNA β‐galactosidase
STAT: signal transducer and activator of transcription
TGF‐β: transforming growth factor‐β
TME: tumor microenvironment
Wnt: wingless‐type MMTV integration site family, member 1
YAP: yes‐associated protein
α‐SMA: alpha‐smooth muscle actin

## 1 Introduction

Pancreatic ductal adenocarcinoma (PDAC) is one of the most aggressive human malignancies, with clinical characteristics of unnoticeable alarming symptoms for early diagnosis, advanced‐stage manifestations, and resistance to chemotherapy [1]. The tumor microenvironment (TME) of PDAC is characterized by prominent desmoplastic stroma with excessive amounts of fibroblasts, collagen, laminin, and fibronectin, which construct a hypovascular microenvironment that impairs drug delivery and increases chemotherapeutic resistance, actively contributing to tumor development and metastasis [2, 3]. Cancer‐associated fibroblasts (CAFs) in the TME play a key role in regulating cancer cell proliferation, invasion, and metastasis [4]. Cancer‐associated fibroblasts can have roles in promoting or retarding tumorigenesis depending on their independence or association with cancer cells [5, 6]. Cancer‐associated fibroblasts could promote cancer cell crosstalk, influence extracellular matrix deposition, and direct the immune system to create a cancer‐promoting environment. By contrast, some fibroblasts inhibit cancer initiation, affect epithelial cell differentiation, and limit cancer cell invasion [7, 8]. Studies have demonstrated more than one CAF population present in the TME of PDAC [9]. Activated PSCs express alpha‐smooth muscle actin (α‐SMA), fibroblast activation protein (FAP), platelet‐derived growth factor‐receptor A (PDGFRA), platelet‐derived growth factor‐receptor B (PDGFRB), and vimentin in the desmoplastic stroma [6, 10, 11]. Earlier studies also reported a positive correlation between CAF infiltration and poor outcomes in patients with cancer, including PDAC [11, 12, 13, 14].

Extracellular vesicles (EVs) are a heterogenous group of cell‐derived membrane‐bound vesicles that vary in size, composition, intracellular biogenesis, and cell of origin. A single cell can deliver a large range of EVs, including small extracellular vesicles (sEV or exosomes; 30–100 nm), microparticles (200 nm–1 μm), and apoptotic bodies, which have different protein profiles due to their different routes of formation [15, 16]. Small extracellular vesicles (or exosomes) released from various cells, act as mediators in intercellular communications by carrying information cargo, such as lipids, proteins, and nucleic acids, into recipient cells [17]. sEVs are enriched with a distinct population of proteins considered sEV markers, such as the transmembrane tetraspanin family (CD9, CD63, and CD81); proteins of endosomal sorting complexes, such as TSG101 and Alix; heat‐shock proteins (HSP70 and HSP90); and major histocompatibility complex class I [18, 19]. Previous studies have shown that sEVs can create an immunosuppressed microenvironment that promotes tumor progression via communication between tumor cells and the surrounding stromal cells [20, 21, 22]. In addition, sEVs can regulate the interaction between tumor cells, immune cells, and fibroblasts resulting in protumorigenic or antitumorigenic responses [23, 24, 25]. Accumulating evidence has also pointed out that sEVs play a significant role in the crosstalk between CAFs and cancer cells and contribute to cancer progression and metastasis [26, 27, 28]. Specific sEVs released from CAFs can be internalized by cancer cells, and sEVs released from cancer cells can induce the transformation of normal fibroblasts into CAFs [29]. In breast cancer, tumor‐secreted sEVs could transfer miR‐9 into normal fibroblasts. The uptake enhances the switch of normal fibroblasts to the CAF phenotype, thus contributing to tumor growth [26]. In melanoma, sEV‐miR‐155 induces the proangiogenic switch of CAFs by inhibiting SOCS1 expression and activating the JAK2/STAT3 signaling pathway [30]. A study has shown that sEVs from PDAC cells promote PSC recruitment through activating the Lin28B/let‐7/HMGA2/PDGFB signaling pathway [31]. However, it is still unclear how the reciprocal effects of tumor‐derived sEVs affect fibroblast activation and tumor progression in PDAC. In this study, we aimed to investigate whether PDAC cell‐derived sEVs are involved in fibroblast activation and to explore the underlying mechanisms that exacerbate PDAC progression and metastasis attributed to the crosstalk between fibroblasts and PDAC cells.

## 2 Materials and methods

### 2.1 Cell lines

Nontransformed human pancreatic ductal epithelial cells (HPDE) from a pancreatic specimen of a 75‐year‐old male [32, 33] (RRID: CVCL_S973, a gift from Dr Tze‐Sing Huang National Health Research Institutes, Taiwan) were grown in keratinocyte serum‐free medium with 0.2 ng·mL^−1^ epidermal growth factor (EGF) and 30 μg·mL^−1^ bovine pituitary extract (Invitrogen Life Technologies, Waltham, MA, USA). Nontransformed human pancreatic acinar‐to‐ductal epithelial‐like cells (HPNE) were purchased from the ATCC (Cat# CRL‐4023, RRID: CVCL_C466). HPNE cells were grown in a medium containing one volume of M3 base medium (INCELL) together with three volumes of glucose‐free DMEM supplemented with 5% FBS, 5.5 mm glucose, 10 ng·mL^−1^ EGF, and antibiotics (penicillin/streptomycin). Pan18 cells were generated from liver metastatic sites of Elas‐CreER; LSL‐Kras^G12D/+^; Trp53^f/+^ male mice (a gift from Dr Chia‐Ning Shen, Genomic Research Center, Academia Sinica, Taipei) [34]. Human embryonic kidney 293T cells (RRID: CVCL_0063) and human pancreatic cancer cells (BxPC‐3) were obtained from the ATCC (Rockville, MD, USA; Cat#CRL‐1687, RRID: CVCL_0186). PDAC patient‐derived PC080 and PC084 cell lines were established as previously described [35]. 293T cells and Pan18 cells were cultured in high‐glucose‐DMEM; BXPC‐3, PC080, and PC084 cells were cultured in RPMI1640 (Gibco, Grand Island, NY, USA). These two media were supplemented with 10% exosome‐depleted fetal bovine serum, penicillin and streptomycin (100 IU·mL^−1^ and 100 μg·mL^−1^, respectively), 1 mm sodium pyruvate, and 1% nonessential amino acids (Gibco). Human pancreatic stellate cells (HPaSteC) were purchased from ScienCell (Catalog #3830, ScienCell Research Laboratories, Inc., Carlsbad, CA, USA) and were cultured in Stellate Cell Medium (SteCM, #5301) supplemented with 2% FBS, 1% Stellate Cell Growth Supplement (SteCGS, #5352), and 1% penicillin/streptomycin mixture (P/S, #0503; ScienCell Research Laboratories, Inc.). Primary cultures of CAFs from human PDAC specimens, including CAF041 and CAF067, were collected from surgically resected PDAC specimens between November 2011 and December 2015 according to our previous study [35], and were maintained in Stellate Cell Medium (SteCM, #5301) supplemented with 2% FBS, 1% Stellate Cell Growth Supplement (SteCGS, #5352), and 1% penicillin/streptomycin mixture (P/S, #0503) (ScienCell Research Laboratories, Inc.). All cells were cultured at 37 °C in a 5% CO_2_ atmosphere and maintained within 3 months of resuscitation from the frozen aliquots with less than 20 passages for each experiment. The cell lines were authenticated by morphology or by Short Tandem Repeat (STR) analysis by analyzing multiple locations within the genome containing short DNA sequence repeats. The resulting DNA profiles were used to confirm the identity and purity of all cell lines through comparison to the STR reference database in the past 3 years. In addition, all cells were regularly checked for mycoplasma infection and cell morphology to keep cells healthy. Human PDAC tissue samples were obtained from patients at the National Taiwan University Hospital (NTUH) with the understanding and written consent of each subject. The study was approved by the National Taiwan University Hospital (NTUH) Institutional Review Board (approval number 201303029RINC and 201411085RINB). The study methodologies conformed to the standards set by the Declaration of Helsinki.

### 2.2 Isolation and culture of mouse primary pancreatic acinar cells and fibroblasts

All mouse primary acinar cells were isolated and cultured as described by Gout et al. [36]. Briefly, 8‐week‐old male C57BL/6 (B6) mouse pancreases were mechanically and enzymatically digested with collagenase IA solution (1× HBSS containing 10 mm HEPES, 200 units·mL^−1^ of collagenase IA, and 0.25 mg·mL^−1^ trypsin inhibitor) to obtain isolated acinar structures. Acini were grown in a medium containing one volume of glucose‐free DMEM and one volume of F12 medium supplemented with 2.5% FBS, 1% penicillin/streptomycin mixture, 0.25 mg·mL^−1^ of trypsin inhibitor, and 25 ng·mL^−1^ of recombinant human EGF. After being cultured on type I collagen‐coated six‐well culture dishes for 6 days, acinar‐to‐ductal‐like cells were trypsinized and replated into another type I collagen‐coated plate for subsequent experiments. Primary fibroblasts from the pancreas of 6‐ to 8‐week‐old B6 mice were isolated and cultured according to a previous report [37].

### 2.3 Separation and characterization of sEVs from cells

The sEVs from the cell‐conditioned medium of HPDE, BXPC‐3, PC080, PC084, Pan18, and mouse pancreatic acinar cells were collected as previously described [38]. The size distribution and concentration of the isolated sEVs were determined as described previously [38].

### 2.4 Protein extraction and western blot analysis

Total proteins were extracted from cultured cells in a lysis buffer containing 50 mm Tris–HCl, 1% NP‐40, 150 mm NaCl, 0.1% SDS, 1 mm PMSF, 1 mm Na_3_VO_4_, and 1 μL protease inhibitor cocktail (Sigma‐Aldrich, Inc., St. Louis, MO, USA). Protein concentrations were determined by the BCA assay kit (Thermo, Carlsbad, CA, USA) with bovine serum albumin as standard. Identical amounts of protein lysates were subjected to 10–12% SDS/polyacrylamide gels and transferred to the polyvinylidene fluoride membrane (Pall Life Sciences, Glen Cove, NY, USA). The membranes were probed with antibodies specific for sEV‐enriched markers, Alix (ab186429), Flotillin‐1 (ab133497), TSG101 (ab125011), CD9 (ab92726) (Abcam, Cambridge, UK), and CD81 (sc‐166 029; Santa Cruz, Dallas, TX, USA); antibodies against Calnexin (#2679; Cell Signaling, Danvers, MA, USA), ezrin (ab40839), α‐SMA (ab7817), PDGFRA (ab203491), PDGFRB (ab32570), fibroblast activation protein (FAP) (ab207178), Active YAP‐1 (ab205270), YAP‐1 (ab52771) (Abcam), STAT1 (#14994), phospho‐STAT1 (pY701) (#7649), STAT2 (#72604), phospho‐STAT2 (#4441; Cell Signaling), STAT3 (#610189; BD Biosciences, Franklin Lakes, NJ, USA), phospho‐STAT3 (pY705; #9131), STAT5 (#94205), phospho‐STAT5 (pY694; #4322), STAT6 (#5397), phospho‐STAT6 (pY641; #9361), PI3K (#4257), phospho‐PI3K (pY458/pY199; #4228), AKT (#4691), phospho‐AKT (pS473; #4060) (Cell Signaling, Danvers, MA, USA), Rab27a (17817‐1‐AP), and Rab27b (13412‐1‐AP) (Proteintech Group Inc, Rosemont, IL, USA). Antibodies against GAPDH (GTX100118; GeneTex, Irvine, CA, USA) were used as an internal control (Table S1). The membranes were exposed using enhanced chemiluminescence reagents (PerkinElmer, Waltham, MA, USA) for the HRP‐coupled secondary antibodies and were analyzed using the BioSpectrum 810 Imaging System (UVP, Upland, CA, USA). Protein levels were detected as the integrated area (pixels) of the band intensities using densitometry analysis with image j software (Bethesda, MD, USA). The numerical values for protein band intensities were corrected with the values for the GAPDH bands.

### 2.5 RNA extraction and quantitative real‐time PCR (qRT‐PCR)

Total RNA was isolated from HPaSteC cell lines using TRIzol reagent (Life Technologies, Gaithersburg, MD, USA) and cDNA was synthesized using Maxima First Strand cDNA Synthesis Kit for qRT‐PCR (Thermo Fisher Scientific, Carlsbad, CA, USA) to produce a template suitable for PCR. The PCR reactions were run on an Eppendorf Mastercycler Nexus Thermal Cycler (Eppendorf AG, Hamburg, Germany). For Col1a1, Col1a2, Col3a3, and Cal12a1 mRNA detection, reverse‐transcription reactions were performed with GAPDH as an internal reference. We use the following primer sequences for PCR reaction: 5′‐CTG CTG GAC GTC CTG GTG AA‐3′ (forward) and 5′‐ACG CTG TCC AGC AAT ACC TTG AG‐3′ (reverse) for Col1a1 expression; 5′‐GAG GGC AAC AGC AGG TTC ACT TA‐3′ (forward) and 5′‐TCA GCA CCA CCG ATG TCC AA‐3′ (reverse) for Col1a2 expression; 5′‐TTG AAG GAG GAT GTT CCC ATC T‐3′ (forward) and 5′‐ACA GAC ACA TAT TTG GCA TGG TT‐3′ (reverse) for Col3a1 expression; 5′‐CAA AGG AGG CAA TAC TCT CAC AG‐3′ (forward) and 5′‐GAA GGT GCT TCA ACA TCG TCT‐3′ (reverse) for Col12a1 expression; and 5′‐GAA GGT GAA GGT CGG AGT‐3′ (forward) and 5′‐GAA GAT GGT GAT GGG ATT TC‐3′ (reverse) for GAPDH expression. qRT‐PCR analysis was used to detect Col1a1, Col1a2, Col3a3, and Cal12a1 using Maestro GreenEvaGreen qPCR Master Mix (Maestrogen, Hsinchu, Xiangshan, Taiwan R.O.C.), respectively, according to the manufacturer's instructions on an Applied Biosystems QuantStudio 5 real‐time PCR system. The expression level was defined based on the threshold cycle, and relative expression levels were calculated as ▵▵*C*_t_ after normalization with reference control.

### 2.6 Plasmids and transfection

For gene knockdown experiments, shRNA clones for shRab27a (TRCN0000100577), shRab27b (TRCN0000100425), shEZR (TRCN0000062459 and TRCN00000380178), and their control pLKO.1‐shLacZ (ASN0000000004) were obtained from the National RNAi Core Facility (Academia Sinica, Taiwan). The plenti6.3/v5‐dest‐EZR plasmid was generated by inserting full‐length cDNA (ezrin: NM_003379) into the plenti6.3/v5‐dest vector. For transfection of the other plasmids, cells were transiently transfected with 5 μg of plasmids using Lipofectamine 3000 from Invitrogen according to the manufacturer's protocol. siRNA‐ezrin (cat# L‐017370‐00‐0010), siRNA‐STAT3 (cat# L‐03544‐00‐0010), siRNA‐Yes‐associated protein (YAP)‐1 (cat# L‐012200‐00‐0010), and the nontargeting siRNA (nonspecific siRNA; NS; cat# D‐001810‐10‐05) were obtained from Dharmacon (Lafayette, LA, USA; Tables S2 and S3).

Transfection was performed using TransIT‐X2 Transfection Reagent (Mirus, Madison, WI, USA) according to the manufacturer's instructions. For lentivirus production, 293T cells were co‐transfected with pMD.G, pCMV▵R8.91, and Lentiviral transfer plasmids using the Polyjet transfection reagent (SignaGen Lab, Ijamesville, MA, USA). We harvested the culture medium containing lentivirus 48 h after transfection and removed the cells from the culture medium by centrifugation at 500 ***g*** for 5 min and filtered it through a 0.45‐μm filter. The lentiviral stocks in the medium containing polybrene (8 μg·mL^−1^) were used to infect the target cells for 6 h.

### 2.7 Transwell migration and invasion assays

Transwell migration and invasion assays were performed using a Transwell insert plate (PET membrane, 8‐μm pore size, SPL 36224, Korea). The transwell invasion assay was conducted after coating the insert membrane with 5 mg·mL^−1^ Matrigel (Corning Matrigel Membrane Matrix, cat# 356234), whereas the transwell migration assay was conducted without coating. HPaSteC migration assay: HPaSteCs (2 × 10^4^ cells per well) treated with sEV (5 μg·mL^−1^) were seeded in the upper chamber in serum‐free medium, and 600 μL of medium containing 2% FBS was added to the lower well. Cells were plated in the top chamber and incubated for 24 h to allow the cells to attach. Cancer cell invasion assay: HPaSteC (1 × 10^5^ cells per well) was seeded in the lower chamber, which was filled with 600 μL of medium. PDAC cells (5 × 10^4^ PDAC cells) were seeded into the upper chamber. After 24 h of incubation, migrated/invading cells were fixed on the membrane with 70% ethanol for 60 min at 4 °C, washed twice with PBS, and before the interiors of the inserts were cleaned with wet cotton swabs. Cells were stained with a cell stain solution (0.1% crystal violet, 20% methanol) and visualized under an inverted microscope (200× magnification; Olympus Corporation, Hachioji, Tokyo, Japan). Images were then counted using an analytical imaging station software package (Imaging Research, Ontario, Canada).

### 2.8 PDAC cell proliferation assay

HPaSteC (2 × 10^4^) were seeded in the upper well of a 24‐well transwell chamber (PET membrane, 0.4‐μm pore, SPL 36224, Korea) and were treated with 5 μg·mL^−1^ sEVs (sEVs from HPDE, HPNE, BXPC‐3, PC080, and PC084 cells) 1 day prior to co‐culture. For co‐culture, PDAC cells (BXPC‐3, PC080, and PC084 cells; 1 × 10^4^) were seeded in the lower well of the transwell chamber. Stellate cell medium was used as the growth medium in both compartments. After 48‐h of incubation, cancer cells in the lower well were fixed in 70% (v/v) ethanol for 60 min at 4 °C, cells were then stained with the cell stain solution (0.1% crystal violet, 20% methanol) and observed under the light microscope. Images were then counted using an analytical imaging station software package (Imaging Research).

### 2.9 Soft agar assay

The soft agar colony formation assay was performed by seeding PDAC cells (5 × 10^4^) and HPaSteC (1 × 10^5^) in a layer of 0.4% agar/complete growth medium over the other layer of 0.8% agar/complete growth medium in the wells of a 12‐well plate. Cultures were maintained in a humidified 37 °C incubator. On day 5 after seeding, the colony‐forming efficiency was quantified under a light microscope. Colonies whose size was quantified by the diameter of each colony were counted and analyzed if larger than 50 μm.

### 2.10 Animal studies

Animal experiments were approved by the Institutional Animal Care and Utilization Committee of Academia Sinica, Taipei, Taiwan (IACUC:14‐05‐709). Mice were maintained in a specific pathogen‐free animal facility at 20 ± 2 °C with a 12/12 h light/dark cycle and had free access to water and standard laboratory chow diet. All animals were monitored for abnormal tissue growth or ill effects according to the AAALAS guidelines and euthanized if the excessive deterioration of health was observed. To assess the effect of the loss of sEVs on the reprogramming of fibroblasts in the tumor, we implanted either 5 × 10^2^ Rab27a/b knockdown or 1 × 10^2^ WT Pan18 cells into the pancreas of 6‐week‐old C57BL/6JNarl (B6) mice (from Taiwan National Laboratory Animal Center). On day 30, all mice were euthanized and xenograft tumors were collected. To study the role of human PDAC cell‐derived sEVs in activating fibroblasts, 6‐week NOD/SCID/IL2Rγ^null^ (NSG) male mice (Genomic Research Center, Academia Sinica, Taipei) were retro‐orbitally injected with sEVs (corresponding to a total protein content of 5 μg) daily of 1 week. Next, 5 × 10^5^ or 1 × 10^6^ GFP‐LUC‐tagged PC080 cancer cells, which were resuspended in 25 μL Matrigel (Corning Inc., Corning, NY, USA), were injected orthotopically into the pancreas. After cancer cell injection, the mice were injected with sEVs (corresponding to a total protein content of 5 μg) every alternate day for 1 week. After 21 days, all mice were euthanized and xenograft tumors were collected. To study liver metastasis, intra‐splenic injection of 5 × 10^4^ GFP‐LUC‐tagged Pan18 and 1 × 10^5^ mouse fibroblast cells were resuspended in 100 μL PBS and injected at the inferior pole of the spleen of 6‐week‐old mice using a 29G needle. After 14 days, the weight and size of the pancreatic tumor in each mouse were analyzed and liver metastases were assessed using bioluminescence IVIS imaging (PerkinElmer).

### 2.11 Immunohistochemical (IHC) staining

Immunohistochemistry was performed on formalin‐fixed, paraffin‐embedded sections using the Leica Bond RX autostainer (Leica Microsystems, Buffalo Grove, IL, USA). After dewaxing and rehydration, samples underwent antigen retrieval at pH6 (ER1)/pH9 (ER2) for 20–30 min, and the diluent was used for protein blocking. α‐SMA Ab (ER2 30 min, 1 : 200, catalog no. NB600‐531; Novus Biologicals, Abingdon, Oxon, UK), PDGFRA Ab (ER2 20 min 1 : 200, catalog no. ab203491), PDGFRB Ab (ER2 20 min, 1 : 200, catalog no. ab32570), and FAP Ab (ER1 20 min 1 : 100, catalog no. ab207178; Abcam) were detected with the Bond Polymer Refine Detection (Leica Microsystems), followed by counterstaining with hematoxylin and bluing reagent (Leica Microsystems). For IHC data analysis, slides were scanned at 40× magnification using Aperio Digital Pathology Slide Scanners; high‐power images (40× magnification) were randomly selected and analyzed by leica aperio imagescope digital slide viewer v9.1.19.1568.

### 2.12 Masson trichrome staining

Collagen staining was performed using the Trichrome Stain Kit (Connective Tissue Stain; ab150686, Abcam) according to the manufacturer's protocol. To quantitatively evaluate trichrome‐stained fibers in each group, representative slides per mouse were chosen, and at least five light microscopy images (40× magnification) were obtained from each slide; lastly, the percentages of stained areas were calculated using leica aperio positive software (Aperio ImageScope v12.3).

### 2.13 Statistical analysis

Except for the animal studies, all experiments were repeated at least 2 or 3 times (indicated as *n* or *N*). Data were presented as the mean ± standard deviation (SD) from repeated independent experiments. Differences between various treatment groups were assessed using the Student's *t* test. Between‐group differences were considered significant at *P* < 0.05. Data analyses were performed using graphpad prism Ver. 5.02 (San Diego, CA, USA).

## 3 Results

### 3.1 PDAC cell‐derived sEVs induce fibroblast activation

**Fig. 1:**
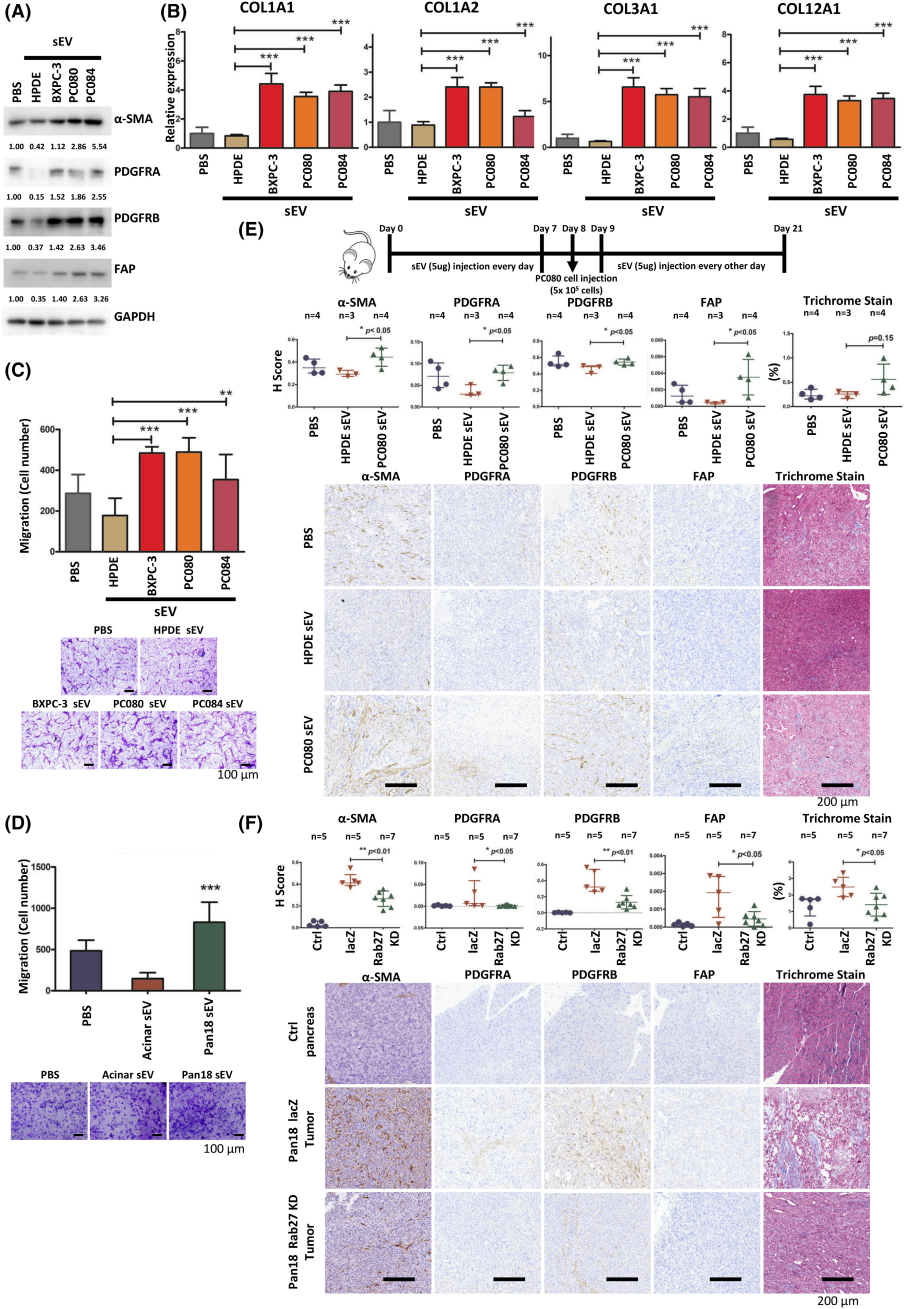
PDAC cell‐derived sEVs induce fibroblast activation. (A) Results of western blotting showing expressions of α‐SMA, FAP, PDGFRA, and PDGFRB in PSCs (HPaSteC) treated with 5 μg·mL^−1^ of PDAC cell‐ or HPDE cell‐derived sEVs for 48 h. GAPDH was used as control. Numerical values for protein band intensities are shown below the gels. The values were quantitated by densitometry and normalized to GAPDH. *N* (number of replicates) = 3. (B) qRT‐PCR analysis of Col1a1, Col1a2, Col3a1, and Cal12a1 in HPaSteC treated with PDAC cell‐ or HPDE cell‐derived sEVs for 48 h. GAPDH was used as an internal control. Values are mean ± SD (*n* (number of cases) = 3). *N* = 3. Level of significance was determined using the Student's *t* test (***P < 0.001). (C) Representative images and the quantified bar chart of Transwell migration assays showing the HPaSteC treated with 5 μg·mL^−1^ of PDAC cell‐ or HPDE cell‐derived sEVs for 24 h. Scale bar, 100 μm. Values are mean ± SD. *N* = 3. Level of significance was determined using the Student's *t* test (**P < 0.01 and ***P < 0.001). (D) Representative images and the quantified bar chart of Transwell migration assays showing the mouse fibroblasts treated with 5 μg·mL^−1^ of Pan18 cell‐ or Acinar cell‐derived sEVs for 24 h. Scale bar, 100 μm. Values are mean ± SD. *N* = 3. Level of significance was determined using the Student's *t* test (***P < 0.001). (E) Schematic illustration of animal study setup and time course. PC080‐derived sEVs or HPDE‐derived sEVs were administered every day for 1 week before injection of PC080 cells in NSG mice, followed by administration every other day until day 21. Representative photomicrographs and the dot chart of Trichrome staining and Immunohistochemistry (IHC) staining of α‐SMA, FAP, PDGFRA, and PDGFRB positive fibroblasts in mouse pancreatic cancer tissues. Each dot represents the datum of a single mouse. Scale bar, 200 μm. 40× magnification. Data presented as mean ± SD. Level of significance was determined using the Student's *t* test (*P < 0.05). *N* = 1. (F) Representative photomicrographs and the dot chart of Trichrome staining and IHC staining of activated fibroblast markers α‐SMA, FAP, PDGFRA, and PDGFRB in normal pancreatic tissues and orthotopic Pan18 cells tumor tissues (Pan18 lacz: Pan18 cells‐shRNA control vectors, Pan18 Rab27 KD: Pan18 cells‐Rab27a/b knockdown) in C57BL/6 mice. Scale bar, 200 μm. 40× magnification. Data presented as mean ± SD. Level of significance was determined using the Student's *t* test (*P < 0.05 and **P < 0.01). *N* = 1. HPaSteC, human pancreatic stellate cells; PDAC, pancreatic ductal adenocarcinoma; PSCs, pancreatic stellate cells; sEVs, small extracellular vesicles.

To further investigate the role of human PDAC cell‐derived sEVs in activating fibroblasts, we treated human pancreatic stellate cells (HPaSteC) with PDAC cell‐derived sEVs, compared with HPDE cell‐derived sEVs. The purified sEVs (exosomes) from PDAC cell lines were characterized by western blotting with sEV‐enriched markers, including Alix, Flotillin‐1, CD9, and CD81 (Fig. S1A), transmission electron microscopy (Fig. S1B), and nanoparticle tracking analysis (Fig. S1C). Western blot analysis showed increased expression of α‐SMA, PDGFRA, PDGFRB, and FAP in fibroblasts treated with PDAC cell‐derived sEVs (Fig. 1A). Fibroblasts treated with PDAC cell‐derived sEVs induced the expression of extracellular matrix genes, COL1A1, COL1A2, COL3A1, and COL12A1, as measured by RT‐qPCR (Fig. 1B). In Transwell assays, PDAC cell‐derived sEVs enhanced HPaSteC migration compared with HPaSteC treated with HPDE cell‐derived sEVs (Fig. 1C). Similarly, mouse PDAC cell (Pan18)‐derived sEVs enhanced migration of mouse fibroblasts compared to those treated with acinar cell‐derived sEVs (Fig. 1D). Furthermore, we implanted PC080 cells into the pancreas of NSG mice and treated mice with HPDE‐ or PC080‐derived sEVs (Fig. 1E). We found an increase in α‐SMA(+), PDGFRA(+), PDGFRB(+), and FAP(+) fibroblasts in orthotropic tumors treated with PC080‐derived sEVs compared to those treated with HPDE‐derived sEVs (Fig. 1E). To assess whether reduction in sEVs secretion affects the activation of fibroblasts in the tumor microenvironment, we compared Pan18‐shRab27a/b cells with Pan18‐shLacz cells implanted into the pancreas of C57BL/6J mice because Rab27a and Rab27b are critical for vesicle trafficking (Kosaka et al., 2013) and depletion of Rab27a/b by shRNA reduce their sEVs secretion (Fig. S1D,E). On day 30, all mice were euthanized and tumors were collected to establish paraffin‐embedded (FFPE) blocks for IHC analysis. We found a decrease in α‐SMA(+), PDGFRA(+), PDGFRB(+), and FAP(+) fibroblasts in orthotropic tumors formed from Pan18‐shRab27a/b cells compared with those formed from Pan18‐shLacZ cells (Fig. 1F). These results suggested that PDAC cell‐derived sEVs induced fibroblast activation.

### 3.2 Fibroblasts activated by PDAC cell‐derived sEVs increase proliferation, invasion, and colony formation of PDAC

**Fig. 2:**
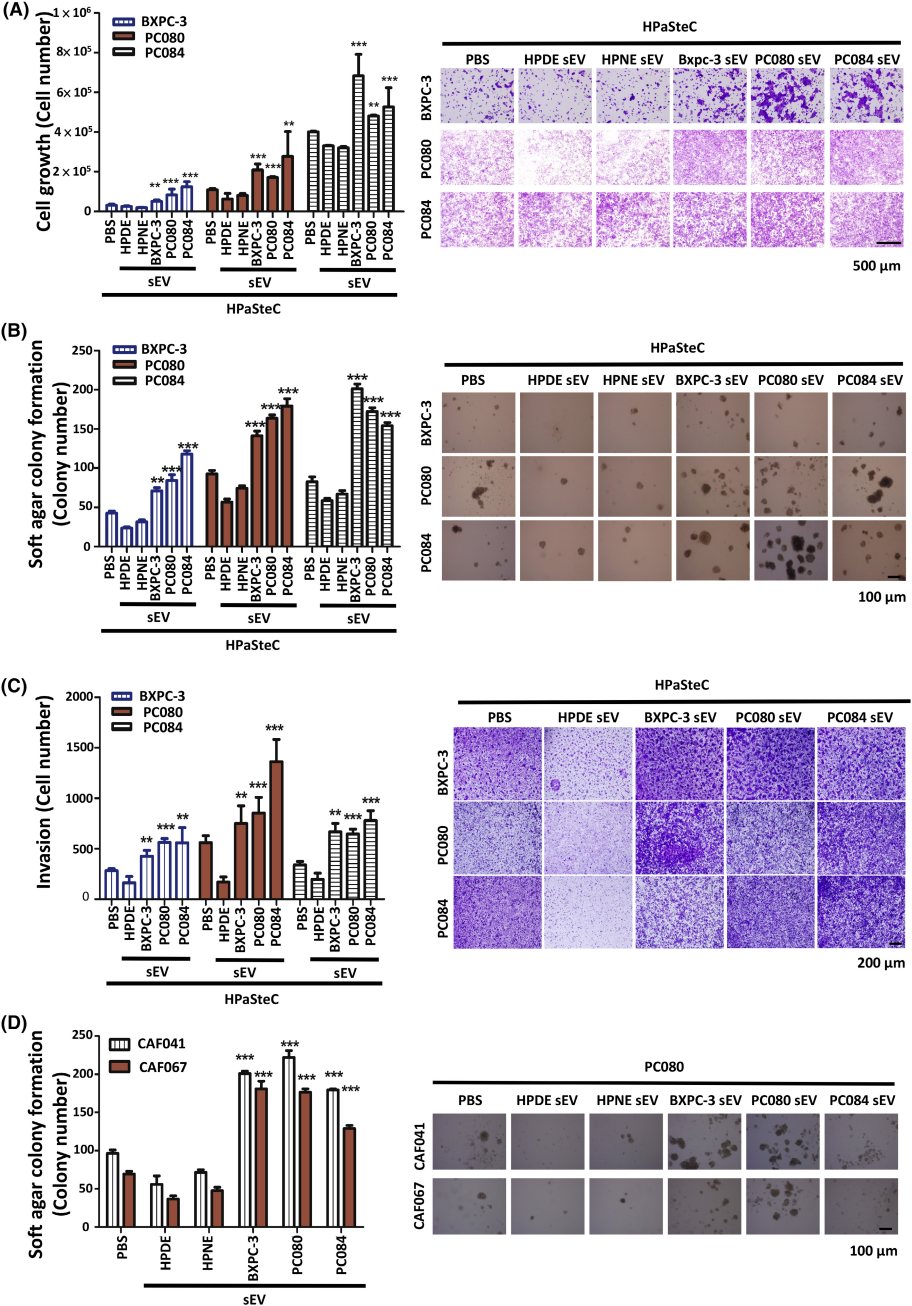
Fibroblasts activated by PDAC cell‐derived sEVs increase PDAC cell proliferation, invasion, and colony formation. (A) Representative images and the quantified bar chart of PDAC cells (BXPC‐3 cells, PC080 cells, and PC084 cells) proliferation co‐cultured with HPaSteC treated with PDAC cell‐ or HPDE/HPNE cell‐derived sEVs for 48 h. Scale bar, 500 μm. Values are mean ± SD (*n* = 3). *N* = 2. (B) Representative images and the quantified bar chart of soft agar colony formation of PDAC cells co‐cultured with HPaSteC treated with 5 μg·mL^−1^ of PDAC cell‐ or HPDE/HPNE cell‐derived sEVs for 5 days. Scale bar, 100 μm. Values are mean ± SD (*n* = 3). *N* = 2. (C) Representative images and the quantified bar chart of Transwell invasion assays of PDAC cells co‐cultured with HPaSteC treated with PDAC cell‐ or HPDE cell‐derived sEVs for 24 h. Scale bar, 200 μm. Values are mean ± SD (*n* = 3). *N* = 2. (D) Representative images and the quantified bar chart of soft agar colony formation of PDAC cells co‐cultured with cancer‐associated fibroblasts (CAFs)‐CAF041 and CAF067 treated with 5 μg·mL^−1^ of PDAC cell‐ or HPDE/HPNE cell‐derived sEVs for 5 days. Scale bar, 100 μm. Values are mean ± SD (*n* = 3). *N* = 2. Level of significance was determined using the Student's *t* test (**P < 0.01 and ***P < 0.001).

To address the potential biological effects of activated fibroblasts on pancreatic cancer cells, we analyzed PDAC cell proliferation by co‐culturing human PDAC cells (BXPC‐3, PC080, or PC084) and HPaSteC, which were pretreated with PDAC cell‐derived sEVs or nontransformed pancreatic cell (HPDE or HPNE)‐derived sEVs for 2 days. On day 2, we found that HPaSteCs treated with PDAC cell‐derived sEVs instead of nontransformed pancreatic cell‐derived sEVs increased PDAC cell proliferation (Fig. 2A). In addition, HPaSteC treated with PDAC cell‐derived sEVs also significantly triggered the formation of soft agar colonies and invasion of BxPC‐3, PC080, and PC084 cells (Fig. 2B,C). Similarly, primary CAFs isolated from human PDAC tissues and treated with PDAC cell‐derived sEVs also promoted the formation of soft agar colonies of PC080 cells (Fig. 2D). These results demonstrated that fibroblasts activated by PDAC cell‐derived sEVs increased PDAC progression in *in vitro* models.

### 3.3 Knockdown of PDAC cell‐derived sEV‐EZR inhibits fibroblast activation

**Fig. 3:**
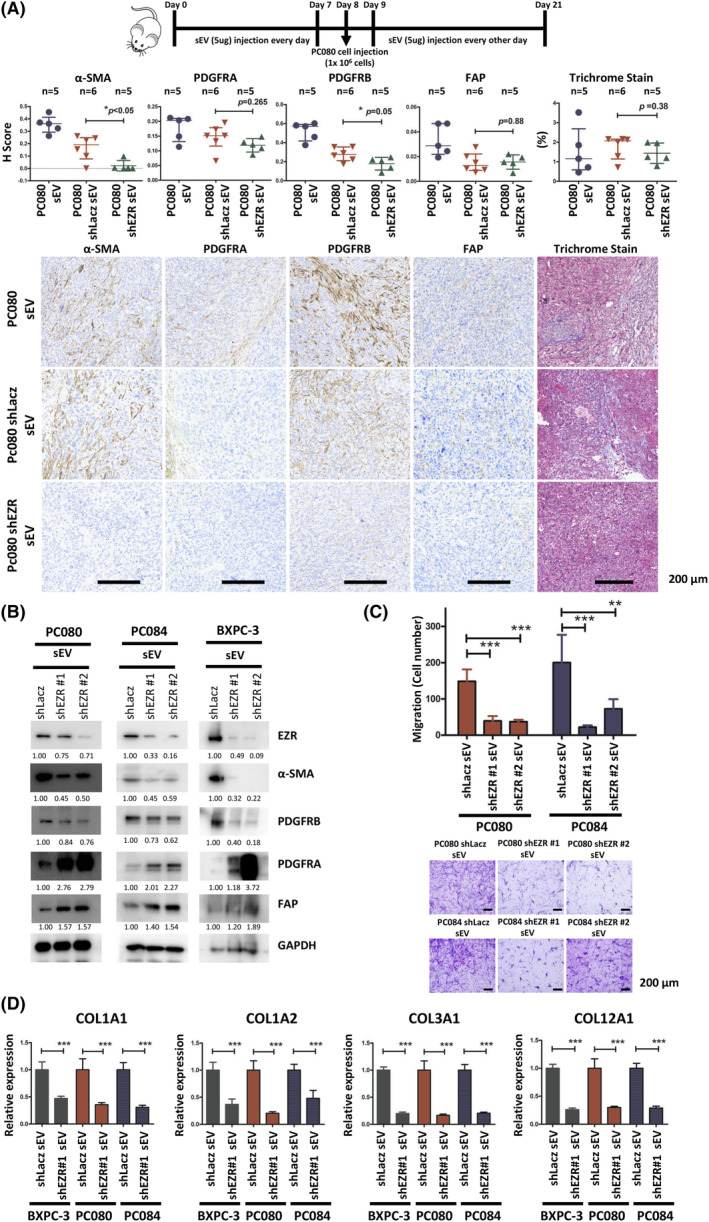
PDAC cell‐derived sEV‐EZR activates fibroblasts. (A) Schematic illustration of animal study setup and time course. PDAC cell‐derived sEVs (PC080‐shLacz sEVs/shEZR‐sEVs) were administered every day for 1 week before PC080 cells injection in NSG mice, followed by administration on every other day until day 21. Representative photomicrographs and the dot chart of Trichrome staining and IHC staining of α‐SMA, FAP, PDGFRA, and PDGFRB positive fibroblasts in mouse pancreatic cancer tissues. Each dot represents the datum of a single mouse. Scale bar, 200 μm. 40× magnification. Data presented as mean ± SD. Level of significance was determined using the Student's *t* test (*P < 0.05). *N* = 1. (B) Results of western blotting showing expressions of EZR, α‐SMA, FAP, PDGFRA, and PDGFRB in HPaSteC treated with 5 μg·mL^−1^ sEVs derived from BXPC‐3, PC080, or PC084 with shLacz sEVs/shEZR‐sEVs for 48 h. GAPDH was used as a protein loading control. Numerical values for protein band intensities are shown below the gels. The values were quantitated by densitometry and normalized to GAPDH. *N* = 2. (C) Representative images and the quantified bar chart of Transwell migration assays of HPaSteC treated with 5 μg·mL^−1^ sEV derived from PC080 or PC084 shLacz sEVs/shEZR‐sEVs for 24 h. Scale bar, 200 μm. Values are mean ± SD (*n* = 3). *N* = 2. Level of significance was determined using the Student's *t* test (**P < 0.01 and ***P < 0.001). (D) qRT‐PCR analysis of Col1a1, Col1a2, Col3a1, and Cal12a1 in HPaSteC treated with 5 μg·mL^−1^ sEVs derived from BXPC‐3, PC080 or PC084 with shLacz sEVs/shEZR‐sEVs for 48 h. GAPDH was used as an internal control. Values are mean ± SD (*n* = 3). *N* = 2. Level of significance was determined using the Student's *t* test (***P < 0.001). shEZR, short hairpin RNA ezrin; shLacz, short hairpin RNA β‐galactosidase (negative control hairpin).

In our previous study, we found abundant expression of ezrin (EZR) in sEVs extracted from pancreatic cancer cell lines and in the plasma of PDAC [38]. EZR knockdown in sEVs derived from BXPC‐3, PC080, and PC084 cells was performed using EZR shRNA and confirmed by western blotting (Fig. S2A). We found a decrease in α‐SMA(+) and PDGFRB(+) fibroblasts in orthotropic tumors treated with PC080‐shEZR‐derived sEVs compared with those treated with PC080‐shLacz‐derived sEVs (Fig. 3A). Western blotting showed a decrease in α‐SMA and PDGFRB expression in HPaSteCs treated with PDAC‐shEZR cell‐derived sEVs, compared with HPaSteCs treated with PDAC‐shLacz‐derived sEVs (Fig. 3B). Conversely, PDAC‐EZR OE (EZR expression vector)‐derived sEVs increased the expression of α‐SMA and PDGFRB in HPaSteCs (Fig. S2B,C). Furthermore, Transwell assays and RT‐qPCR analysis showed that HPaSteC treated with PDAC‐shEZR cell‐derived sEVs suppressed HPaSteC migration and decreased the expression of COL1A1, COL1A2, COL3A1, and COL12A1 in fibroblasts, compared with those treated with PDAC‐shLacz‐derived sEVs (Fig. 3C,D). These results demonstrate that PDAC cell‐derived sEV‐EZR activated fibroblasts and upregulated the expression of α‐SMA, PDGFRB, and the extracellular matrix in fibroblasts.

### 3.4 Fibroblasts activated by PDAC cell‐derived sEV‐EZR exacerbate PDAC metastasis

**Fig. 4:**
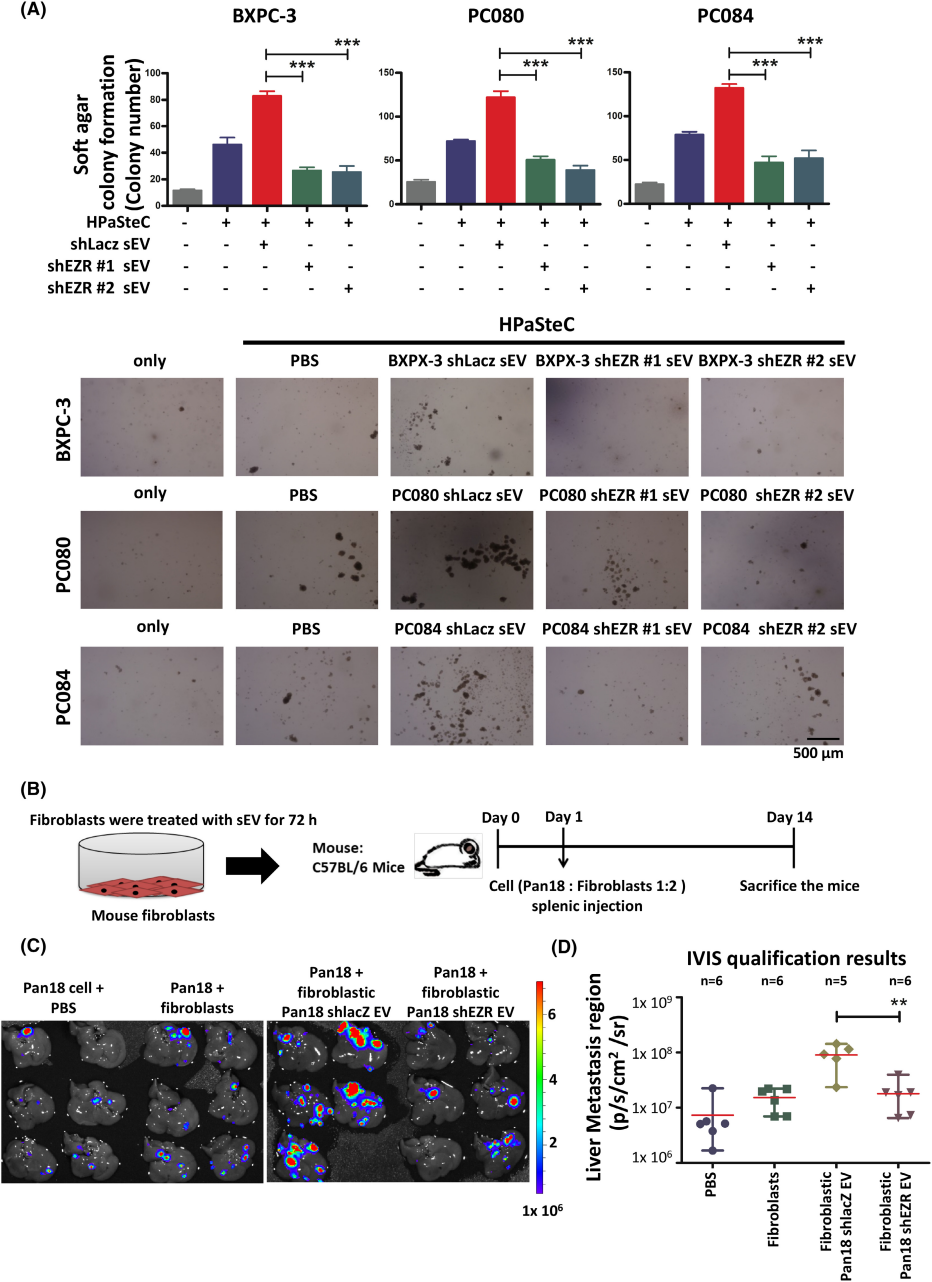
PDAC cell‐derived sEV‐EZR exacerbates cancer metastasis to the liver. (A) Representative images and the quantified bar chart of soft agar colony formation of PDAC cells co‐cultured with HPaSteC treated with 5 μg·mL^−1^ of PDAC‐shLacz or shEZR cell‐derived sEVs for 5 days. Scale bar, 500 μm. Values are mean ± SD (*n* = 3). *N* = 2. Level of significance was determined using the Student's *t* test (***P < 0.001). (B) Schematic illustration of animal study setup and time course for the *in vivo* mouse liver metastasis model. Pan18 and mouse fibroblasts were co‐injected into the spleen of 6‐week‐old male C57BL/6J mice. Approximately 14 days postinjection, the mice were found with metastatic liver tumors derived from the injected PDAC cells. The total number of mice examined in each group was six (*n* = 6). (C) IVIS images showed liver metastasis on day 14. (D) The bioluminescence photon counts in the region of liver metastasis (*n* = 6) were measured by IVIS software. The bioluminescent signal (pseudocolor) was recorded as photons per second (p/s). Values are mean ± SD. The red horizontal line indicates mean. Level of significance was determined using the Student's *t* test (**P < 0.01). *N* = 1.

Next, we examined whether fibroblasts treated with PDAC cell‐derived sEVs would affect tumor growth and metastasis of PDAC via sEV‐EZR. PDAC cells co‐cultured with fibroblasts treated with PDAC‐shEZR cell‐derived sEVs showed significantly lower colony formation than those treated with PDAC‐shLacz cell‐derived sEVs (Fig. 4A). In animal experiments, we injected 5 × 10^4^ Pan18 GFP‐luc cells and 1 × 10^5^ mouse fibroblast cells into the exteriorized spleens of 6‐week‐old male C57BL/6J mice and monitored tumor colonization by bioluminescence imaging. Approximately 14 days postinjection, the mice developed metastatic liver tumors derived from the injected PDAC cells (Fig. 4B). The amount of liver metastasis formed by Pan18 and fibroblasts treated with Pan18‐shEZR‐derived sEVs was less than that formed by Pan18 and fibroblasts treated with Pan18–shLacz (Fig. 4C,D). These data suggest that fibroblasts treated with PDAC cell‐derived sEV‐EZR promote tumor growth and metastasis.

### 3.5 Ezrin increases both α‐SMA and PDGFRB expression in fibroblasts through the STAT3/YAP‐1 signal pathways

**Fig. 5:**
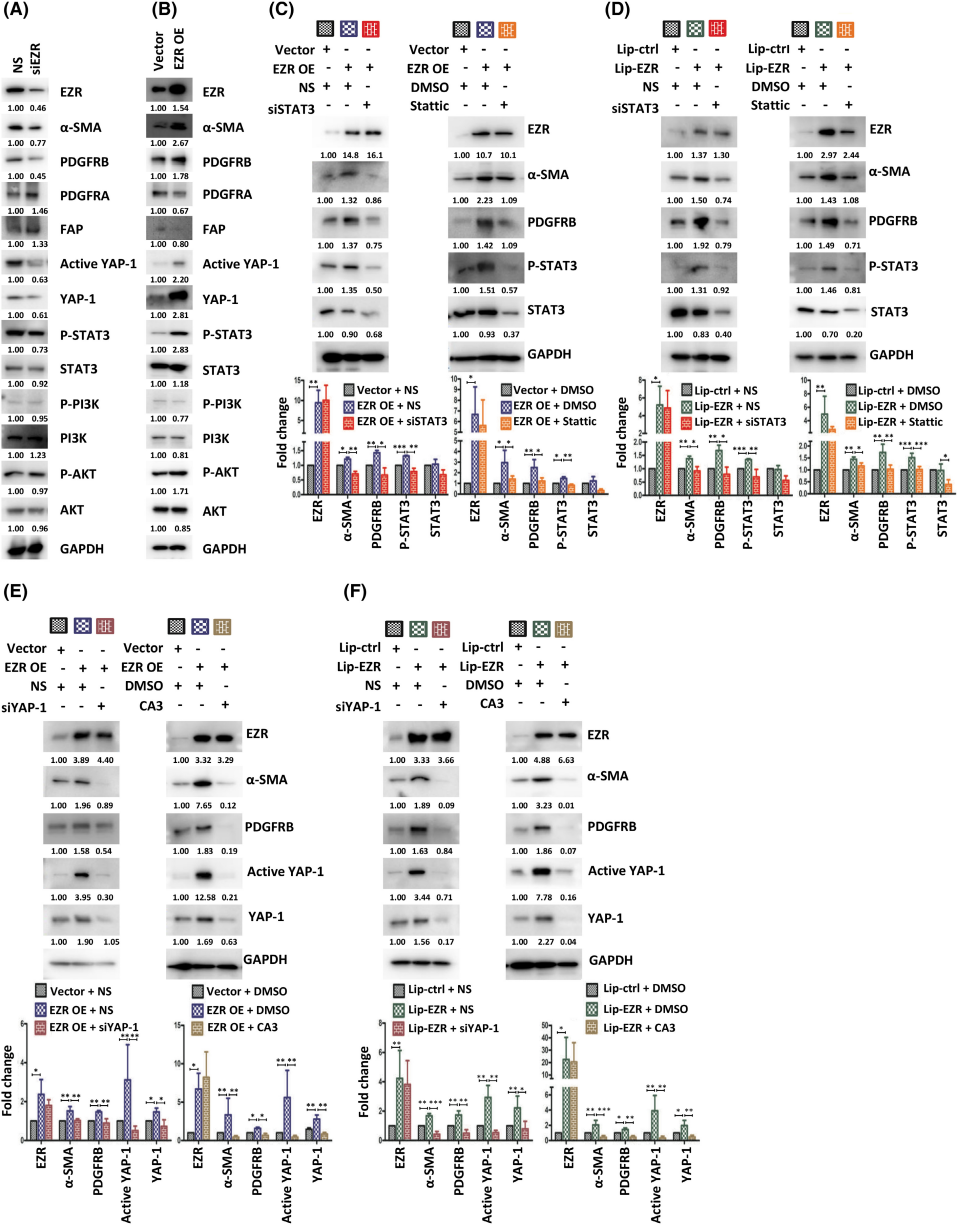
PDAC cell‐derived sEVs induces α‐SMA and PDGFRB expression in fibroblasts via EZR/STAT3 and EZR/YAP‐1 signaling pathways. (A) Results of western blotting of EZR, α‐SMA, PDGFRB, PDGFRA, FAP, YAP‐1, active YAP‐1, STAT3, AKT, PI3K, and their phosphorylation in HPaSteC transfected with siRNA‐EZR or NS (nonspecific siRNA) for 48 h. *N* = 2. (B) Expressions of EZR, α‐SMA, PDGFRB, PDGFRA, FAP, YAP‐1, active YAP‐1, STAT3, AKT, PI3K, and their phosphorylation in HPaSteC transfected with EZR OE (EZR expression vector) or Vec (control vector) for 48 h. The numerical values for protein band intensities were corrected with the values for the loading control GAPDH bands. *N* = 2. (C) Results of western blotting of EZR, α‐SMA, PDGFRB, STAT3, and phospho‐STAT3 in HPaSteC transfected with EZR OE or Vec and then transfected with siRNA‐STAT3 or NS (left) or treated with 5 μm Stattic (STAT3 inhibitor; right). Values are mean ± SD (*n* = 3). Level of significance was determined using the Student's *t* test (*P < 0.05, **P < 0.01, ***P < 0.001). (D) Results of western blotting showing the expressions of EZR, α‐SMA, PDGFRB, STAT3, and phospho‐STAT3 in HPaSteC treated with Lip‐EZR (Liposomal‐EZR) or Lip‐ctrl (Liposomal‐control) and then transfected with siRNA‐STAT3 or NS (left) or treated with 5 μm Stattic (right). Values are mean ± SD (*n* = 3). Level of significance was determined using the Student's *t* test (*P < 0.05, **P < 0.01, ***P < 0.001). (E) Results of western blotting showing the expressions of EZR, α‐SMA, PDGFRB, YAP‐1, and active YAP‐1 in HPaSteC transfected with EZR OE or Vec and then transfected with siRNA‐YAP‐1 or NS (left) or treated with 100 nm CA3 (YAP‐1 inhibitor; right). Values are mean ± SD (*n* = 3). Level of significance was determined using the Student's *t* test (*P < 0.05 and **P < 0.01). (F) Results of western blotting showing the expressions of EZR, α‐SMA, PDGFRB, YAP‐1, and active YAP‐1 in HPaSteC treated with Lip‐EZR or Lip‐ctrl and then transfected with siRNA‐YAP‐1 or NS (left) or treated with 100 nm CA3 (right). Values are mean ± SD (*n* = 3). Level of significance was determined using the Student's *t* test (*P < 0.05, **P < 0.01, ***P < 0.001). GAPDH was used as control. Numerical values for protein band intensities are shown below the gels. The values were quantitated by densitometry and normalized to GAPDH.

**Fig. 6:**
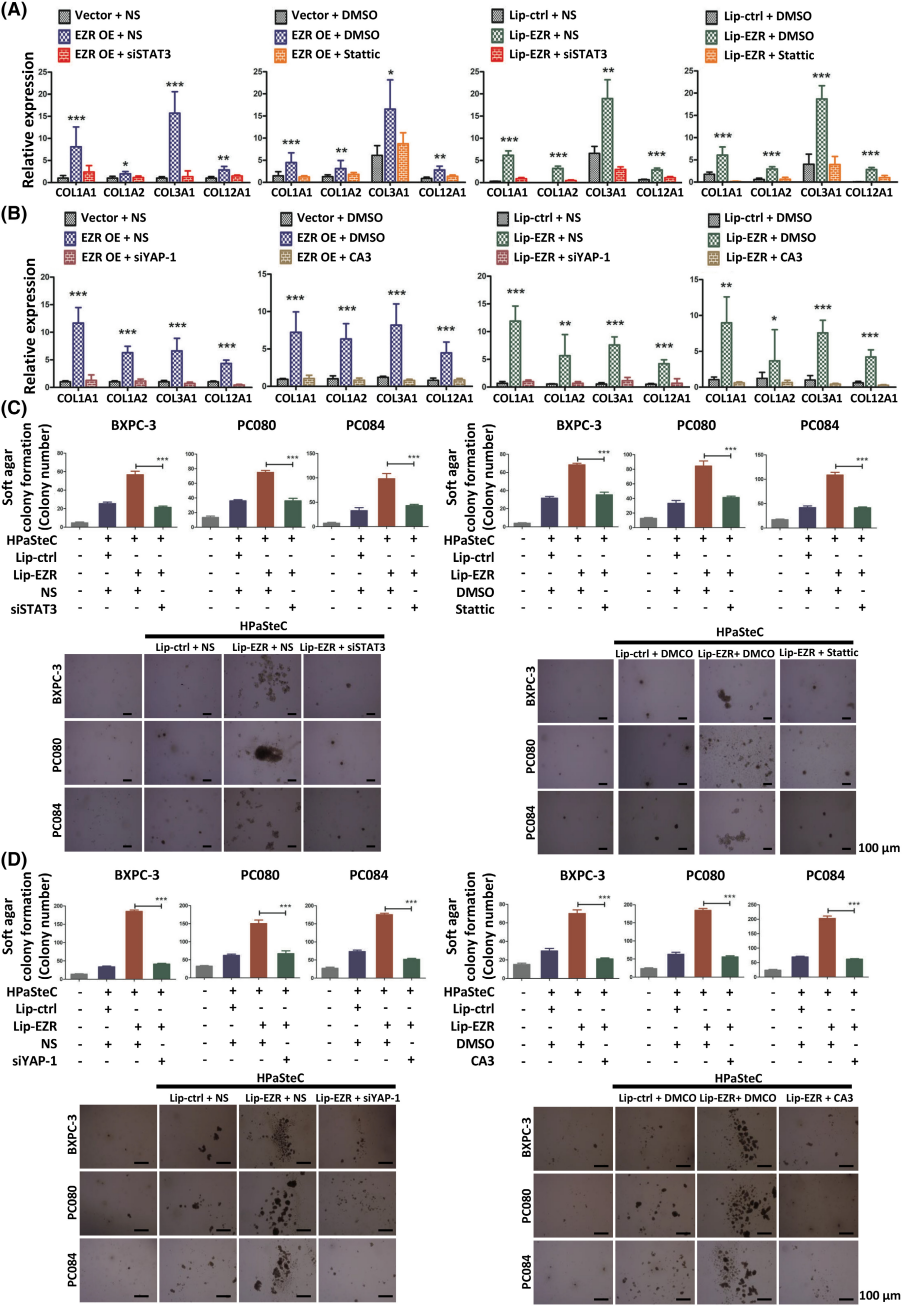
Inhibition of EZR/STAT3 or EZR/YAP‐1 signaling pathways abrogates the EZR‐induced activation of Fibroblasts. (A) qRT‐PCR analysis of expressions of Col1a1, Col1a2, Col3a1, and Cal12a1 in HPaSteC, whose EZR was overexpressed (transfected with EZR OE or treated with Lip‐EZR) and then transfected with siRNA‐STAT3 or treated with 5 μM Stattic. GAPDH was used as an internal control. Values are mean ± SD (*n* = 3). *N* = 2. Level of significance was determined using the Student's *t* test (*P < 0.05, **P < 0.01, ***P < 0.001). (B) qRT‐PCR analysis of the expressions of Col1a1, Col1a2, Col3a1, and Cal12a1 in HPaSteC whose EZR was overexpressed (transfected with EZR OE or treated with Lip‐EZR) and then transfected with siRNA‐ YAP‐1 or treated with 100 nm CA3. GAPDH was used as an internal control. Values are mean ± SD (*n* = 3). *N* = 2. Level of significance was determined using the Student's *t* test (*P < 0.05, **P < 0.01, ***P < 0.001). (C) Representative images and the quantified bar chart of soft agar colony formation of PDAC cells co‐cultured with HPaSteC treated with Lip‐EZR and then transfected with siRNA‐STAT3 or treated with 5 μm Stattic for 5 days. Scale bar, 100 μm. Values are mean ± SD (*n* = 3). *N* = 2. Level of significance was determined using the Student's *t* test (***P < 0.001). (D) Representative images and the quantified bar chart of soft agar colony formation of PDAC cells were co‐cultured with HPaSteC treated with Lip‐EZR and then transfected with siRNA‐YAP‐1 or treated with 5 μm CA3 for 5 days. Scale bar, 100 μm. Values are mean ± SD (*n* = 3). *N* = 2. Level of significance was determined using the Student's *t* test (***P < 0.001).

To investigate how EZR regulates the expression of α‐SMA, PDGFRA, PDGFRB, and FAP, we silenced EZR in HPaSteC cells using siRNA, which strongly reduced the protein expression of α‐SMA and PDGFRB (Fig. 5A). Overexpression of EZR in HPaSteCs increased α‐SMA and PDGFRB protein expression (Fig. 5B). PI3K/Akt, NF‐κB, Wnt, JAK/STAT, FAK, and YAP are known to be downstream signaling transduction pathways of EZR [39, 40]. We examined YAP‐1, PI3K/Akt, and JAK/STAT signaling by overexpressing or silencing EZR in fibroblasts. As shown in Fig. 5A,B, EZR overexpression in HPaSteC significantly induced STAT3 phosphorylation and YAP‐1 expression. Silencing EZR by siRNA in HPaSteCs reduced STAT3 phosphorylation and YAP‐1 expression. α‐SMA and PDGFRB expression in HPaSteC was reduced due to inhibition of STAT3 or YAP‐1 expression by STAT3 or YAP‐1‐specific siRNA or inhibitor in fibroblasts, which were transfected with EZR expression plasmid or treated with Liposomal‐EZR [prepared using EZR recombinant protein (ab132942, Abcam) and Pierce Protein Transfection Reagent (Cat# 89850, Thermo Fisher) according to the manufacturer's instructions] (Fig. 5C–F). STAT3 inhibitor (Stattic)/siSTAT3 and YAP‐1 inhibitor (CA3)/siYAP‐1 (Table S4) also reduced COL1A1, COL1A2, COL3A1, and COL12A1 expression in HPaSteCs, whose EZR was overexpressed (Fig. 6A,B). Inhibition of STAT3 and YAP‐1 in HPaSteC also decreased PDAC cell colony formation in the soft agar co‐culture assay (Fig. 6C,D). Knockdown of STAT3 or YAP‐1 in fibroblasts had no significant effect on EZR protein levels in HPaSteC (Fig. 5C–F). These results indicate that PDAC cell‐derived sEVs activate HPaSteC through the EZR/STAT3 and EZR/YAP‐1 signaling pathways.

## 4 Discussion

Fibroblasts play a significant role in modulating tumor growth and metastasis in the tumor microenvironment. CAF infiltration is associated with not only lymphatic invasion and lymph node metastasis but also poor outcomes and shorter survival of PDAC [11, 12, 14, 41]. The exact origin of CAFs and the mechanism by which quiescent fibroblasts develop into CAFs remain unclear. However, based on current evidence, a considerable number of CAFs originate from PSC surrounding cancer cells in PDAC [4]. PSCs are resident cells of the pancreas and quiescent in healthy individuals [42]. In chronic pancreatitis or PDAC, PSCs are activated and contribute to inflammatory and pro‐fibrogenic reactions [42]. Activated PSCs express high levels of α‐SMA and secrete excessive amounts of ECM proteins, leading to desmoplasia in chronic pancreatitis and PDAC [42, 43]. CAFs modulate cancer metastasis through the synthesis and remodeling of the extracellular matrix (ECM) and the production of growth factors, which influence angiogenesis, tumor mechanics, drug access, and therapy responses [44, 45]. CAFs can activate cancer cells via multiple mechanisms in a dose‐dependent manner [46]. Fibroblasts can be activated through mechanical contact with cancer cells, cancer cell‐derived sEVs, and/or cytokines. For example, transforming growth factor‐β (TGF‐β) signaling [47], Interleukin‐6 (IL‐6), and PDGF are known fibroblast‐activating factors [48]. sEVs released by cancer cells can transfer proteins, RNA, and miRNAs to stromal fibroblasts, thereby contributing to their recruitment and activation [50, 51]. Many studies have reported that sEVs released from CAFs can be internalized by cancer cells and contribute to cancer progression and metastasis [29]. Correspondingly, sEVs released by cancer cells can induce the conversion of normal fibroblasts into CAFs. Pang et al. [51] reported that PDAC cell‐derived microvesicles containing miR‐155, which had been taken up by recipient fibroblasts, could convert normal fibroblasts into CAFs via the downregulation of TP53INP1. In breast cancer and melanoma, cancer cell‐derived sEVs containing different miRNAs have been found to convert normal fibroblasts into CAFs, thereby contributing to tumor progression [26, 30]. These results indicate that sEVs play a crucial role in the activation of fibroblasts and interactions between cancer cells and fibroblasts. α‐SMA and PDGFRB are both markers of activated fibroblasts that play crucial roles in desmoplasia [52]. Previous studies have also reported that α‐SMA and PDGFRB expression in fibroblasts has pro‐metastatic effects mostly through matrix remodeling in invasive breast cancer [53]. In this study, we showed that sEVs released from PDAC cells upregulated the expression of α‐SMA, PDGFRA, PDGFRB, and FAP and induced excessive production of collagen (COL1A1, COL1A2, COL3A1, and COL12A1) in fibroblasts (Fig. 1). Specifically, the transfer of molecular cargo from PDAC cells to stromal cells via sEVs is a key regulator of CAF activation.

sEVs released from cancer cells are packed with unique information cargo such as lipids, proteins, and nucleic acids that can be transferred to recipient cells. Regarding the mechanism whereby PDAC cell‐derived sEVs regulate fibroblast activation, we found that EZR in PDAC cell‐derived sEVs plays an important role in the regulation of α‐SMA and PDGFRB expression in fibroblasts, and that knockdown of EZR in PDAC cell‐derived sEVs attenuates the expression of α‐SMA and PDGFRB in fibroblasts. Ezrin is a member of the ERM (ezrin/radixin/moesin) family, which regulates membrane remodeling [54]. EZR is involved in many cellular processes, including cell adhesion, migration, determination of cell shape, cell proliferation, and morphogenesis, in response to extracellular cues [55]. Aberrant EZR expression is associated with poor prognosis and metastasis in various cancers including PDAC [57, 58]. Previous studies have shown that EZR promoted pancreatic cancer cell proliferation, migration, and invasion through upregulating the FAK/AKT signaling pathway [58]. The CAF plays a vital role in the complex process of tumor metastasis [59]. Activated fibroblasts exhibit a change in morphology and can acquire smooth muscle‐like properties with increased contractility, motility, proliferation, and a stellate morphology [60]. Therefore, the ezrin protein is of particular interest in fibroblasts as it plays a role in regulating cell morphology through its interaction with the membrane and actin cytoskeleton. Studies have found that co‐culture of CAF with the conditioned medium of BRCA1‐deficient human breast cancer cells HCC1937 can increase the expression level of ezrin mRNA in CAF by 30‐fold, which helps tumor cells to metastasize and invade [61]. These results suggest that ezrin plays an essential role in the CAF to promote tumor cell metastasis. In this study, we have shown that PDAC cell‐derived sEVs with ezrin activate normal fibroblasts, and reciprocally, activated fibroblasts promote tumor invasion and proliferation in *in vitro* transwell assays and co‐culture experiments. Moreover, in our animal experiments, the amount of liver metastasis of PDAC in Pan18 cells co‐injected with fibroblasts treated with shLacz‐derived sEVs was larger than that in Pan18 cells co‐injected with fibroblasts treated with Pan18‐shEZR‐derived sEVs. We demonstrated that PDAC cells secrete EZR‐rich sEVs to induce α‐SMA and PDGFRB expression in fibroblasts, exacerbating tumor progression and metastasis in PDAC. We observed the reversed expression of PDGFRA and PDGFRB in fibroblasts treated with sEV‐EZR. This is in line with the previous studies that the expression of PDGFRB generally increases, while that of PDGFRA decreases in fibroblasts [63, 64]. Previous studies have shown that the α‐SMA and PDGFRB expression in fibroblast had pro‐metastatic effects through matrix remodeling [53]. We found that EZR in PDAC‐derived sEVs plays an important role in the regulation of α‐SMA and PDGFRB expression in fibroblasts, and that knockdown of EZR in PDAC‐derived sEVs attenuates the expression of α‐SMA and PDGFRB in fibroblasts. This indicates that sEV‐EZR can regulate CAF subtypes that express both α‐SMA and PDGFRB.

The molecular mechanisms by which EZR participates in tumorigenesis are not well characterized [39], nor is the role of sEV‐EZR in fibroblast activation. Previous studies have shown that the phosphorylation of STAT3 and YAP is involved in stabilizing the activated phenotype of CAFs [30, 65]. STAT3 is a latent transcription factor that plays a role in regulating fibroblast function. STAT3 signaling also transmits the profibrotic effects of TGF‐β on fibroblasts and that targeted inhibition of JAK2 or STAT3 ameliorates fibroblast activation and fibrosis [65]. The melanoma cell‐secreted sEVs could activate CAFs through the elevation of the phosphorylation levels of JAK2 and STAT3 in CAFs [30]. Furthermore, YAP‐1 overexpression augmented the proliferation, migration, expression of collagen, fibronectin, and Hippo/YAP in fibroblasts. Similarly, YAP‐1 enhanced the proliferation, migration, and transition of cultured lung fibroblasts into myofibroblasts [66]. Ezrin has a direct role in the regulation of Yap in skin fibroblast proliferation. Further, ezrin depletion was shown to impair skin fibroblast proliferation through its effect on YAP nuclear translocation [40]. These results show that STAT3 and YAP are key transcriptional factors in regulating fibroblast activation and function. Our study further demonstrated that sEV‐EZR regulates α‐SMA and PDGFRB expression in fibroblasts via the STAT3 or YAP‐1 signaling pathways. Moreover, we found that silencing of STAT3 and YAP‐1 also blocks EZR‐induced α‐SMA and PDGFRB expression and the production of excessive amounts of collagen from fibroblasts. Lastly, we report that the YAP‐1 inhibitor (CA3)/siYAP‐1 could cause a greater decrease in both α‐SMA and PDGFRB expression in EZR‐overexpressing fibroblasts compared with the STAT3 inhibitor (Stattic)/siSTAT3. These findings suggest that YAP‐1 may play a more important role in activating α‐SMA and PDGFRB expression in fibroblasts. More importantly, our results confirmed that PDAC cell‐derived sEV‐EZR modulates the STAT3 and YAP‐1 signaling pathways to promote fibroblast activation, and the activated fibroblasts reciprocally exacerbate PDAC metastasis. Inhibition of EZR/STAT3 or EZR/YAP‐1 signaling pathways abrogates EZR‐induced activation of fibroblasts, and targeting CAFs and desmoplasia might be a promising approach to treat PDAC. Therefore, our study provides evidence that targeting STAT3 or YAP‐1 or EZR inhibitors is a feasible treatment option for PDAC. Although previous studies have shown that fibroblast recruitment is essential for tumor development and that sEVs from PDAC cells can promote PSC recruitment by activating the Lin28B/let‐7/HMGA2/PDGFB signaling pathway [31, 68, 69], further studies are needed to elucidate the mechanisms by which PDAC cell‐derived sEVs recruit fibroblasts to the tumor.

## 5 Conclusion

This is the first study to show that PDAC cell‐derived sEV‐EZR can activate fibroblasts to express α‐SMA and PDGFRB possibly via STAT3 and YAP‐1 signaling pathways, which in turn exacerbate PDAC metastasis. Whether STAT3 and YAP‐1 activation and sEV‐EZR are linked remains to be experimentally verified. Thus, targeting sEV‐EZR may contribute to improve adjuvant PDAC therapies.

## Conflict of interest

The authors declare no conflict of interest.

## Author contributions

Y‐TC and H‐YP involved in conception and design. Y‐TC, H‐YP, C‐MH, S‐CT, Y‐IC, and Y‐MJ involved in the development of methodology. Y‐TC, H‐YP, M‐CC, C‐MH, S‐CT, Y‐IC, and Y‐MJ involved in the acquisition of data (provided animals, acquired and managed patients, provided facilities, etc.). H‐YP, M‐CC, C‐MH, and Y‐TC involved in analysis and interpretation of data (e.g., statistical analysis, biostatistics, and computational analysis). Y‐TC and H‐YP involved in writing—original draft preparation. Y‐TC and C‐MH involved in writing—review and editing. M‐CC, C‐MH, and Y‐TC involved in administrative, technical, or material support (i.e., reporting or organizing data, constructing databases). M‐CC and Y‐TC involved in study supervision. M‐CC and Y‐TC involved in funding acquisition.

## Supporting information


**Fig. S1.** Separation and characterization of sEVs from conditioned medium via ultracentrifugation (UC) and a sucrose density gradient (SDG).
**Fig. S2.** sEV‐EZR regulates α‐SMA and PDGFRB expression in fibroblasts.
**Table S1.** List of antibody information/resource.
**Table S2.** List of siRNA information/resource.
**Table S3.** List of shRNA information/resource.
**Table S4.** List of chemical information/resource.

## Data Availability

All data supporting the findings of this study are available in the paper and Supplementary Information.
