# Supplementary material for: Pancreatic cancer‐derived small extracellular vesical ezrin activates fibroblasts to exacerbate cancer metastasis through STAT3 and YAP‐1 signaling pathways
Source: Mol Oncol. 2023 May 12;17(8):1628–47. doi: 10.1002/1878-0261.13442 (PMC10399719; doi:10.1002/1878-0261.13442)
Supplement: Supplementary file 1 — Fig. S1. Separation and characterization of sEVs from conditioned medium via ultracentrifugation (UC) and a sucrose density gradient (SDG). Fig. S2. sEV‐EZR regulates α‐SMA and PDGFRB expression in fibroblasts. Table S1. List of antibody information/resource. Table S2. List of siRNA information/resource. Table S3. List of shRNA information/resource. Table S4. List of chemical information/resource. [file MOL2-17-1628-s001.pdf]

Supplementary Materials for

Pancreatic cancer-derived small extracellular vesical ezrin activates fibroblasts to exacerbate cancer metastasis through STAT3 and YAP-1 signaling pathways

Yu-Ting Chang*, Hsuan-Yu Peng*, Chun-Mei Hu, Sui-Chih Tien, Yi-Ing Chen, Yung-Ming Jeng, and Ming-Chu Chang
*Co-first authors

Corresponding author
Yu-Ting Chang, M.D., M.S., Ph.D. e-mail: yutingchang@ntu.edu.tw

This file includes:

Supplementary Figures 1 to 2
Supplementary Tables 1 to 4

Supplementary Figure

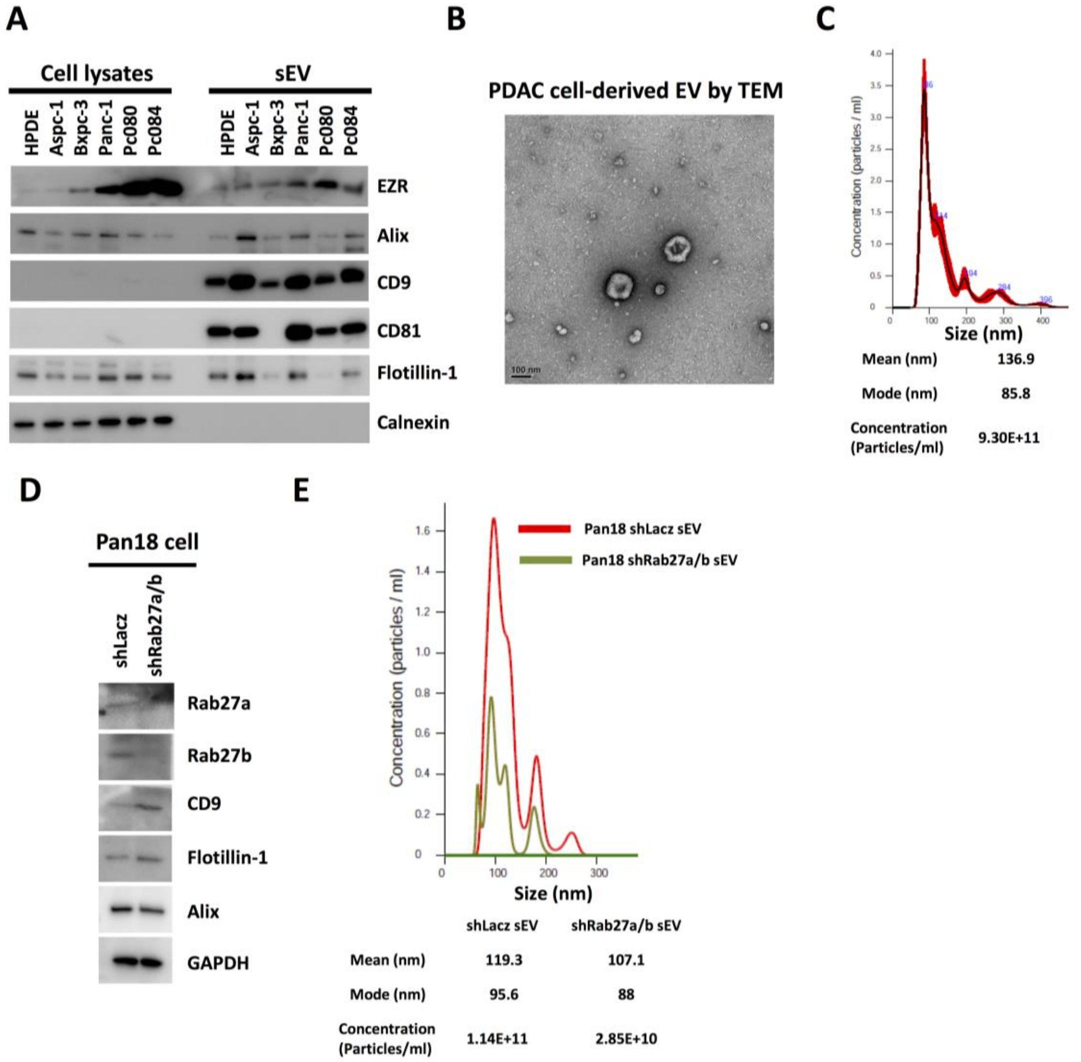

Supplementary Figure 1- Separation and Characterization of sEVs from conditioned medium via ultracentrifugation (UC) and a sucrose density gradient (SDG). A. Western blots of sEV-enriched markers. Abundant Flotillin-1, Alix, CD9, and CD81 are detected in sEVs isolated from conditioned medium by UC-SDG. B. PDAC cell-derived sEVs were observed by TEM. Scale bars are 100 nm. C. EV concentration and size distribution by nanoparticle tracking analysis (NTA, NasoSight 300). D. Western blot analysis of the Rab27a, Rab27b, Flotillin-1, Alix, and CD9 form after transfection of shRab27a/b (Rab27a/b knock down) in Pan18 cells. GAPDH was used as protein loading control. E. NTA of EVs concentrations and size by NasoSight 300 from purified Pan18 shLacz sEV and Pan18 shRab27a/b sEV.

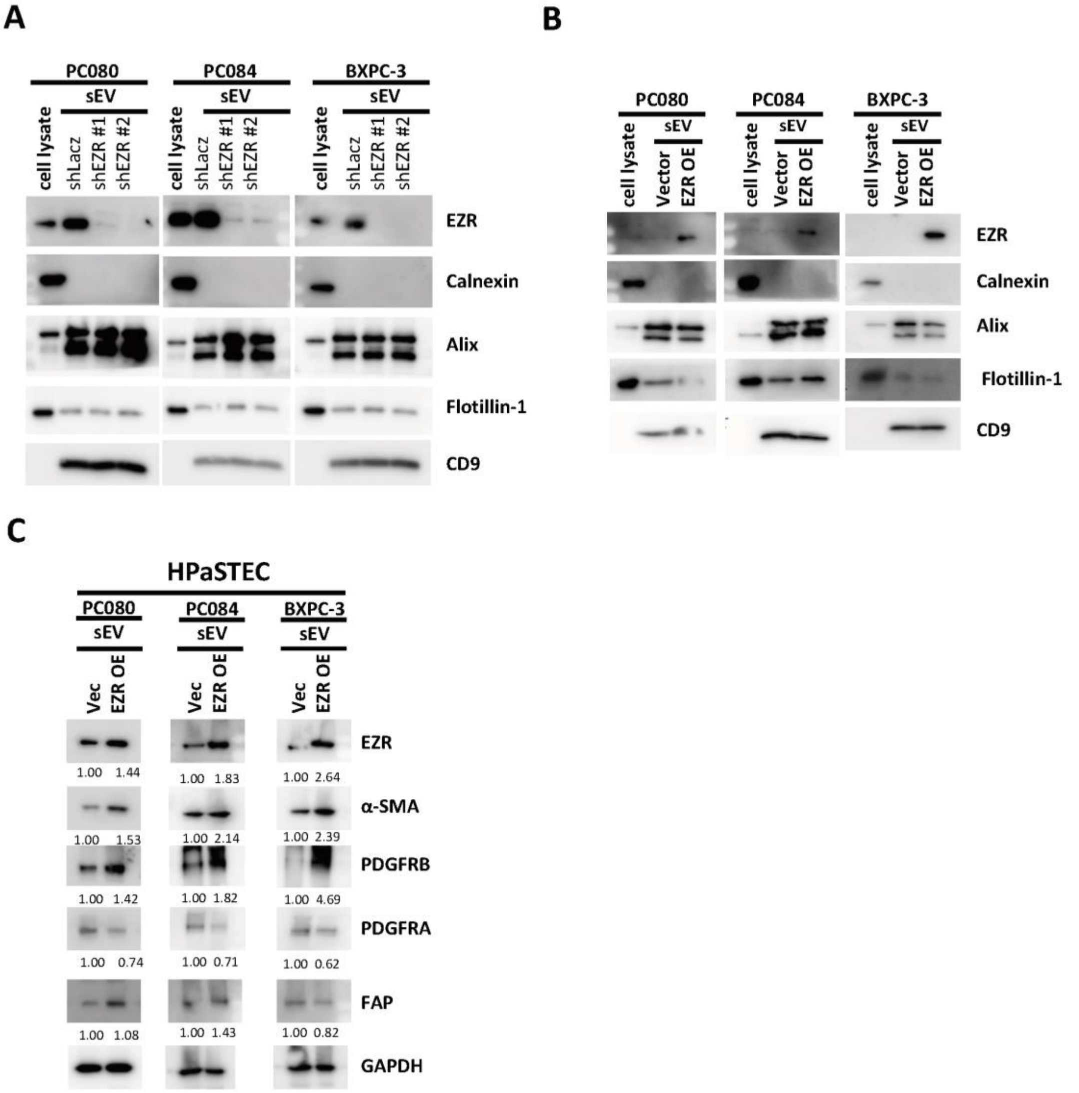

Supplementary Figure 2- sEV-EZR regulates α-SMA and PDGFRB expression in fibroblasts. A. Western blot analysis of expression EZR in BXPC-3, PC080, and PC084-derived sEVs with knockdown EZR by shRNA (#1 and #2). B. Western blot analysis of expression EZR in sEVs derived from BXPC-3-EZR OE, PC080-EZR OE, PC084-EZR OE, BXPC-3-Vector, PC080-Vector, or PC084-Vector. C. Western blotting of expression of EZR, α-SMA, FAP, PDGFRA and PDGFRB in HPaSteC treated with sEVs derived from BXPC-3, PC080, PC084-EZR OE-derived sEVs, BXPC-3, PC080, and PC084-Vector-derived sEVs for 48hrs.

Supplementary Table 1. List of antibody information/resource

Antibody	Identifier	Source
EZR Rabbit monoclonal [EP886Y]	Cat# ab40839, RRID: AB_732275	Abcam
Alix Rabbit monoclonal [EPR15314]	Cat# ab186429, RRID: AB_2754981	Abcam
Flotillin-1 Rabbit monoclonal [EPR6041]	Cat# ab133497, RRID: AB_11156367	Abcam
TSG101 Rabbit monoclonal [EPR7130(B)]	Cat# ab125011, RRID: AB_10974262	Abcam
CD9 Rabbit monoclonal [EPR2949]	Cat# ab92726, RRID: AB_10561589	Abcam
CD81 Mouse monoclonal (B-11)	Cat# sc-166029, RRID: AB_2275892	Santa Cruz Biotechnology
Calnexin Rabbit monoclonal (C5C9)	Cat# 2679, RRID: AB_2228381	Cell Signaling Technology
α-SMA mouse monoclonal [1A4]	Cat# ab7817, RRID: AB_262054	Abcam
PDGFR alpha Rabbit monoclonal [EPR22059-270]	Cat# ab203491, RRID: AB_2892065	Abcam
PDGFR beta Rabbit monoclonal [Y92]	Cat# ab32570, RRID: AB_777165	Abcam
FAP Rabbit monoclonal [EPR20021]	Cat# ab207178, RRID: AB_2864720)	Abcam
Active YAP1 Rabbit monoclonal [EPR19812]	Cat# ab205270, RRID: AB_2813833	Abcam
YAP-1 Rabbit monoclonal [EP1674Y]	Cat# ab52771, RRID: AB_2219141)	Abcam
alpha-Smooth Muscle Actin Rabbit polyclonal	Cat# NB 600-531, RRID: AB_521547	Novus
GAPDH Rabbit Polyclonal	Cat# GTX100118, RRID: AB_1080976	GeneTex
Rab27a Rabbit polyclonal	Cat# 17817-1-AP, RRID: AB_2176728	Proteintech
Rab27b Rabbit polyclonal	Cat# 13412-1-AP, RRID: AB_2176732	Proteintech
STAT1 Rabbit monoclonal [D1K9Y]	Cat# 14994,	Cell Signaling

	RRID: AB_2737027	Technology
phospho-STAT1 (Tyr701) Rabbit monoclonal	Cat# 7649, RRID: AB_10950970	Cell Signaling Technology
STAT2 Rabbit monoclonal [D9J7L]	Cat# 72604, RRID: AB_2799824	Cell Signaling Technology
Phospho-Stat2 (Tyr690) Rabbit polyclonal	Cat# 4441, RRID: AB_2198445	Cell Signaling Technology
STAT3 mouse monoclonal [84]	Cat# 610189, RRID: AB_397588	BD Biosciences
Phospho- STAT3 (Tyr705) Rabbit polyclonal	Cat# 9131, RRID: AB_331586	Cell Signaling Technology
STAT5 Rabbit monoclonal [D2O6Y]	Cat# 94205, RRID: AB_2737403	Cell Signaling Technology
phospho-STAT5 Rabbit monoclonal [D47E7]	Cat# 4322, RRID: AB_10544692	Cell Signaling Technology
STAT6 Rabbit monoclonal [D3H4	Cat# 5397, RRID: AB_11220421	Cell Signaling Technology
Phospho- STAT6 (Tyr641) Rabbit polyclonal	Cat# 9361, RRID: AB_331595	Cell Signaling Technology
PI3 Kinase p85 Rabbit monoclonal [19H8]	Cat# 4257, RRID: AB_659889	Cell Signaling Technology
Phospho-PI3 Kinase p85 (Tyr458)/p55 (Tyr199) Rabbit polyclonal	Cat# 4228, RRID: AB_659940	Cell Signaling Technology
AKT (pan) Rabbit monoclonal [C67E7]	Cat# 4691, RRID: AB_915783	Cell Signaling Technology
Phospho-Akt (Ser473) Rabbit monoclonal [D9E]	Cat# 4060, RRID: AB_2315049	Cell Signaling Technology

Supplementary Table 2. List of siRNA information/resource

siRNA	Target Sequence	Identifier	source
Non-targeting siRNA pool	UGGUUUACAUGUCGACUAA,<br>UGGUUUACAUGUUGUGUGA,<br>UGGUUUACAUGUUUUCUGA,<br>UGGUUUACAUGUUUUCCUA,	D-001810-10-05	Dharmacon
EZRIN siRNA pool	GCGCGGAGCUGUCUAGUGA,<br>GCGCAAGGAGGAUGAAGUU,<br>GGAAUCAACUAUUUCGAGA,<br>GCUCAAAGAUAAUGCUAUG,	L-017370-00-0010	Dharmacon
STAT3 siRNA pool	GAGAUUGACCAGCAGUAUA,<br>CAACAUGUCAUUUGCUGAA,<br>CCAACAAUCCCAAGAAUGU,<br>CAACAGAUUGCCUGCAUUG,	L-03544-00-0010	Dharmacon
YAP1 siRNA pool	GCACCUAUCACUCUCGAGA,<br>UGAGAACAAUGACGACCAA,<br>GGUCAGAGAUACUUCUUAA,<br>CCACCAAGCUAGAUAAAGA,	L-012200-00-0010	Dharmacon

Supplementary Table 3. List of shRNA information/resource

shRNA	Target Sequence	Clone ID	source
pLKO.1-shLacZ	CCTAAGGTTAAGTCGCCCTCG	ASN0000000004	National RNAi Core Facility (Taipei, Taiwan)
shEZR #1	CCAGCCAAATACAACTGGAAA	TRCN0000062459	National RNAi Core Facility (Taipei, Taiwan)
shEZR #2	TGATGCCCTTGGACTGAATAT	TRCN00000380178	National RNAi Core Facility (Taipei, Taiwan)
shRab27a	GCTTCTGTTCGACCTGACAAA	TRCN0000100577	National RNAi Core Facility (Taipei, Taiwan)
shRab27b	CCTGAGACAATGTCAAACCAT	TRCN0000100425	National RNAi Core Facility (Taipei, Taiwan)

Supplementary Table 4. List of chemicals information/resource

Chemicals	Target	Identifier	source
Stattic	STAT3	A12733	AdooQ® Bioscience
CA3(CIL56)	YAP-1	S8661	Selleckchem
